# Supplementary material for: A Global Screen for Assembly State Changes of the Mitotic Proteome by SEC-SWATH-MS
Source: Cell Syst. 2020 Feb 26;10(2):133–155.e6. doi: 10.1016/j.cels.2020.01.001 (PMC7042714; doi:10.1016/j.cels.2020.01.001)

P15923 | TFE2_HUMAN | TCF3 BHLHB21 E2A ITF1
Monomer MW [kDa]: 67.6 Monomer expected elution fraction: 44

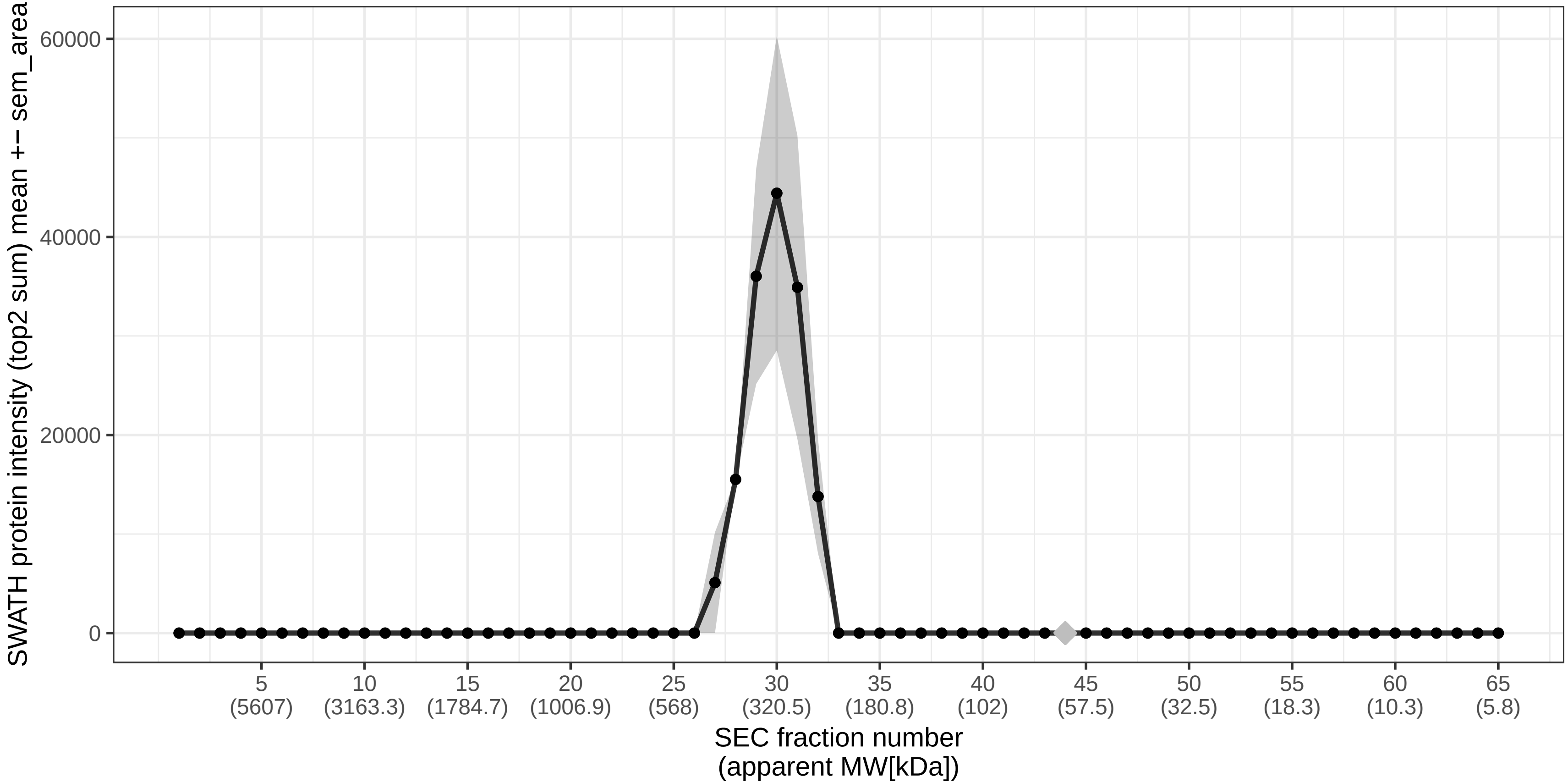

Supplement: Data S1. SEC-SWATH-MS Protein Chromatograms, Related to Figure 1 [file mmc6.zip › SECchrom_P15923_TFE2_HUMAN_TCF3_BHLHB21_E2A_ITF1.pdf]
